# Automatic Identification of Bioprostheses on X-ray Angiographic Sequences of Transcatheter Aortic Valve Implantation Procedures Using Deep Learning

**DOI:** 10.3390/diagnostics12020334

**Published:** 2022-01-27

**Authors:** Laura Busto, César Veiga, José A. González-Nóvoa, Marcos Loureiro-Ga, Víctor Jiménez, José Antonio Baz, Andrés Íñiguez

**Affiliations:** 1 Cardiovascular Research Group, Galicia Sur Health Research Institute (IIS Galicia Sur), 36213 Vigo, Spain; jose.gonzalez@iisgaliciasur.es (J.A.G.-N.); marcos.loureiro@iisgaliciasur.es (M.L.-G.); 2 Cardiology Department, Complexo Hospitalario Universitario de Vigo (SERGAS), Álvaro Cunqueiro Hospital, 36213 Vigo, Spain; victor.alfonso.jimenez.diaz@sergas.es (V.J.); jose.antonio.baz.alonso2@sergas.es (J.A.B.); andres.iniguez.romo@sergas.es (A.Í.)

**Keywords:** angiography, deep learning, segmentation, transcatheter aortic valve implantation (TAVI), U-Net

## Abstract

Transcatheter aortic valve implantation (TAVI) has become the treatment of choice for patients with severe aortic stenosis and high surgical risk. Angiography has been established as an essential tool in TAVI, as this modality provides real-time images required to support the intervention. The automatic interpretation and parameter extraction on such images can lead to significative improvements and new applications in the procedure that, in most cases, rely on a prior identification of the transcatheter heart valve (THV). In this paper, U-Net architecture is proposed for the automatic segmentation of THV on angiographies, studying the role of its hyperparameters in the quality of the segmentations. Several experiments have been conducted, testing the methodology using multiple configurations and evaluating the results on different types of frames captured during the procedure. The evaluation has been performed in terms of conventional classification metrics, complemented with two new metrics, specifically defined for this problem. Those new metrics provide a more appropriate assessment of the quality of the results, given the class imbalance in the dataset. From an analysis of the evaluation results, it can be concluded that the method provides appropriate segmentation results for this dataset.

## 1. Introduction

Transcatheter aortic valve implantation (TAVI) is the procedure for the replacement of the aortic valve in the heart through blood vessels, using catheters to deliver and deploy the bioprosthesis. Due to its numerous advantages, the procedure has been established during the last decade as the main treatment for most patients presenting symptomatic severe aortic stenosis [1]. It has been proved to be more beneficial than medical treatment for inoperable cases, becoming a better alternative to surgery on intermediate or high risk patients [2]. There is also a consensus in the imaging modalities and protocols involved in all stages of TAVI [3], including several different modalities at pre, peri, and post-procedural states [4]. The standard practice uses intra-procedural angiography and pre-implant three-dimensional imaging sources to ensure transcatheter heart valve (THV) correct placement, assessing the position and functioning immediately after deployment and identifying any intra-procedural complication. Thus, angiography has become an important supporting tool during TAVI due to its ability to produce real-time views [5].

Even if the imaging role in TAVI is clearly defined in the procedural guides, the possibility of extracting additional value from images gathered during and after the procedure for its evaluation and the prediction of long-term outcomes is still an open challenge [5]. Despite the maturity of the modality, the automatic interpretation of these images would boost new applications for angiography prior to and during TAVI, facilitating the standardization of the procedure and potentially limiting its complications. Such applications could include the assessment of the optimal deployment, which can be obtained by measuring the depth of the left ventricular edge of the device in the left ventricular outflow tract (LVOT) relative to the annular plane of the aortic valve [6]. Another possible utility is the implant characterization by extracting parameters as the stent tip deflection, the maximum bioprosthesis extension, and LVOT eccentricity [7]. All those applications rely on a prior requirement, which is the precise and automatic identification of the THV. Thus, detecting and tracking the bioprosthesis in angiographic X-ray imaging sequences has several new practical applications [8]. This automatic detection points directly to the hot topic of Artificial Intelligence (AI) in medical imaging [9]. AI has been successfully applied to several cardiovascular imaging modalities [10,11]; however, its use in percutaneous cardiovascular interventions has scarcely been reported [12].

This work presents a way to improve TAVI by the automatic detection of the bioprosthesis on X-ray angiographic sequences. U-Net architecture is proposed for this purpose, testing different hyperparameter configurations and studying their impact on the segmentation results. Such network is trained and tested using an annotated dataset generated by the research group. The validation of any methodology plays an important role in this realm [13], and the task becomes specially complex in class imbalance problems [14], as the one presented in this work. New metrics have been defined specifically for this imbalanced segmentation, which provide a more proper assessment of the quality of the results.

The next section introduces the required background in terms of imaging particularities; Deep Learning and, particularly, U-Net fundamentals; and the metrics used to assess the performance of this kind of systems. Section 3 describes the proposed methodology and its implementation. In Section 4, results of the performed experiments are provided, and the last section includes the discussion and conclusions.

## 2. Background

This section introduces the main parts of the proposed approach: angiographic imaging in TAVI, U-Net architecture, and evaluation metrics suitable to assess the methodology.

### 2.1. Angiographic Imaging in TAVI

**Figure 1:**
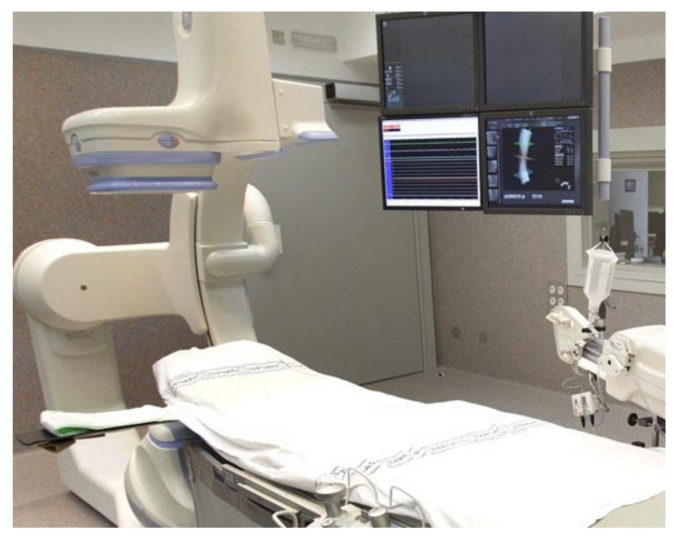
Álvaro Cunqueiro Hospital Cath Lab.

TAVI takes place in a catheterization laboratory, such as the one shown in Figure 1, which includes the C-arm that produces the real-time angiographic sequences during the intervention.

**Figure 2:**
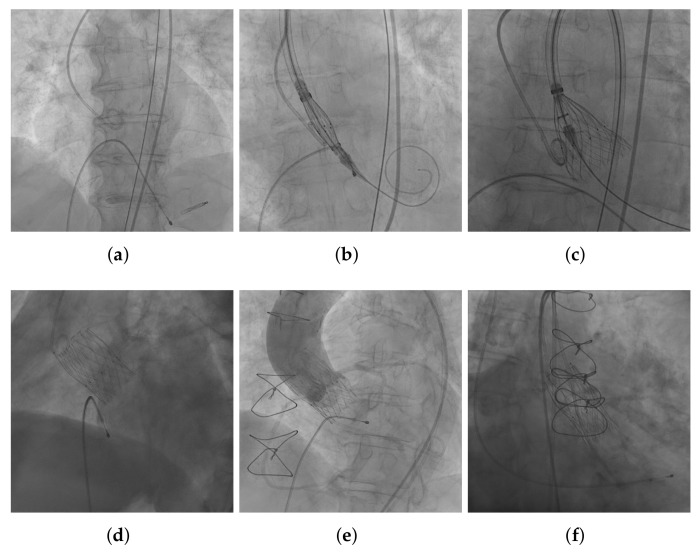
Angiographic frames captured during TAVI. (**a**) Aortic root previous to the device delivery with a pigtail catheter in the non-coronary sinus, and a temporary pacemaker lead in the right ventricle. (**b**) THV loaded in the delivery catheter and positioned in the aortic annulus before deployment. (**c**) First part of the THV deployment in the aortic annulus. (**d**) THV completely deployed in the aortic annulus. (**e**) Functioning assessment of a deployed THV using a contrast agent injection. (**f**) Other elements overlaping the device (previous surgical sutures at the level of the sternum and a temporary pacemaker lead in the right ventricle).

During the procedure, the bioprosthesis is introduced compressed in the catheter, using angiographic views it is guided towards the aortic root, and finally placed on the native valve (Figure 2b). Using angiographic imaging, the optimal plane is identified, which is crucial for the success of the bioprosthesis positioning. Once the device is placed in the appropriate location, it is deployed (Figure 2c), until it is fully expanded (Figure 2d). It is remarkable the great variety of images gathered in different stages of the procedure, as all the examples in Figure 2. An example of an angiographic sequence is provided as a Supplementary Figure S1 (angioseq.gif).

Nowadays, there are several manufacturers that produce different THV models, with different shapes and sizes. However, all of them share a characteristic radiopaque reticular pattern, which is perceivable in the angiographies, independently of the image generation properties or angulations.

### 2.2. U-Net

In a Deep Learning application, two important aspects are the network architecture and the training parameters and strategy.

Concerning architectures, U-Net is a convolutional neural network developed for biomedical image segmentation [15]. It relies on the use of data augmentation, which means that the images presented to the network are modified versions of the original ones, generated on the fly by applying elastic transformations. By using data augmentation, the network does not process the same image twice in the different epochs, enlarging the dataset and aiming to teach the network the desired invariance and robustness properties.

With respect to the training, the stage when the network weights are adjusted in each epoch such that a cost function is optimized, several optimizers and cost functions can be employed. One frequently used cost function is cross-entropy, defined as follows for an image binary classification:

(1) $$CE=-\sum_{i=1}^{N\times M}y_{i}\log\left(p_{i}\right)+\left(1-y_{i}\right)\log\left(1-p_{i}\right),$$

where *i* is an index over all the pixels of an image of size N×M. $y_{i}\in \left\{0,\;1\right\}$ corresponds to the pixel classification in the image annotation, background or foreground respectively. $p_{i}$ is the probability of the pixel *i* to belong to foreground, predicted by the model. Equation (1) can be written as (2):

(2) $$CE=-\sum_{i=1}^{N\times M}\log\left(q_{i}\right),$$

where $q_{i}$ is defined as follows:

(3) $$q_{i}=\left\{\begin{array}{cc} p_{i} & \mathrm{if}\;y_{i}=1\\ 1-p_{i} & \mathrm{otherwise}. \end{array}\right.$$

In situations of highly imbalanced classes, a well-known alternative to cross-entropy is focal loss [16]. Focal loss expression consists in a modification of cross-entropy with a modulating factor ${\left(1-q_{i}\right)}^{\gamma }$ that decreases as the classification confidence increases, where γ≥0 is a tunable *focussing* parameter. In practice, the following α-balanced variant (4) of the focal loss is used.

(4) $$FL=-\sum_{i=1}^{N\times M}\alpha_{i}{\left(1-q_{i}\right)}^{\gamma }\log\left(q_{i}\right),$$

where $\alpha_{i}$ is obtained as follows:

(5) $$\alpha_{i}=\left\{\begin{array}{cc} \alpha & \mathrm{if}\;y_{i}=1\\ 1-\alpha & \mathrm{otherwise}. \end{array}\right.$$

In short, these α and γ parameters repercute in the contribution of each sample to the loss. α∈[0,1] plays a role in the balance of the classes (background and foreground in binary image segmentation), whereas γ affects the weights of correct and wrong classifications.

### 2.3. Evaluation Metrics

The evaluation of the results plays a crucial role in general image segmentation problems, and in medical imaging in particular [13]. Most frequent metrics in binary classification consist in a pixel-wise evaluation that requires a gold standard where an expert determines the pixels on each image corresponding to the target to detect, often by manually drawing it. Comparing the gold standard with the results permits quantitative evaluation and statistics. Some widely used classification metrics are accuracy, precision, and recall [17]. Other common methods are the Receiver Operating Characteristic (ROC) [18] curve analysis, particularly the area under the ROC curve (AUROC), and metrics measuring overlapping areas between finite sample sets, such as Dice coefficient [19] and Jaccard index [20].

A different evaluation approach is measuring the distance between sets of points. One of those is Hausdorff distance [21], which is the greatest of all the distances from a point in one set to the closest point in the other set, defined as:

(6) $$HD\left(G,S\right)=\max_{g\in G}\left(\min_{s\in S}\left(d(g,s)\right)\right),$$

where G and S are gold standard and segmentation result respectively, both binary images, and *G* and *S* are the sets of non-zero elements in these images. *g* and *s* iterate over the points of such sets and d(·) denotes the Euclidean distance between two points.

## 3. Materials and Methods

The methodology followed in this work distinguishes four main blocks. First block is image dataset acquisition and annotation. The second block is the hyperparameter tuning of a U-Net architecture, which allows to select the network configuration. In third place, the training and test of the model using such configuration. Finally, the assessment of the segmentation results obtained by such model. These evaluation results are used by the hyperparameter tuning block for the selection of the configuration that provides the best segmentation results for this dataset among the considered options.

The whole work has been implemented in Python, using Keras [22] and Tensorflow [23] libraries. The training of this kind of networks take advantage of high performance computational infrastructures. In this case, the computations of this work were performed in a GPU cluster owned by the research group, which has three NVIDIA TESLA A100 GPUs.

### 3.1. Image Acquisition and Annotation

The images used in this work belong to a total of 15 patients suffering from severe aortic stenosis. All of the patients have been implanted an Allegra (New Valve Technology AG, Muri, Switzerland) THV of different sizes: 23 mm, 27 mm, or 31 mm. This prosthesis is a self-expandable valve made of a nitinol stent frame and bovine pericardium leaflets with a supra-annular functioning. These cases are consecutive procedures with such implant that took place from June 2019 to January 2020 at the Department of Cardiology at Álvaro Cunqueiro Hospital, Vigo, Spain. The trial protocol was approved by the Spanish national authorities and ethics committees and by institutional research boards at the participating site. Informed consent was obtained and documented for all patients before conducting any study-related procedures.

The TAVI procedures were performed according to the local protocol at the participating site. TAVI images were obtained by conventional fluoroscopy and cine at 15 and 25 fps on AlluraClarity equipment with ClarityIQ technology (Philips, Amsterdam, The Netherlands). During the procedure, unfractionated heparin was used with the goal of an activated clotting time >250 s and the decision to reverse its action with protamine at the end of the procedure were done according to local standard.

Several sequences have been captured from each patient and from different projections. All the sequences contain between 25 and 100 frames, and it is guaranteed that all of them contain at least a complete heartbeat. Each frame is of 512 × 512 pixels, with a pixel resolution of 8 bits and a pixel spacing of 0.34 mm.

**Figure 3:**
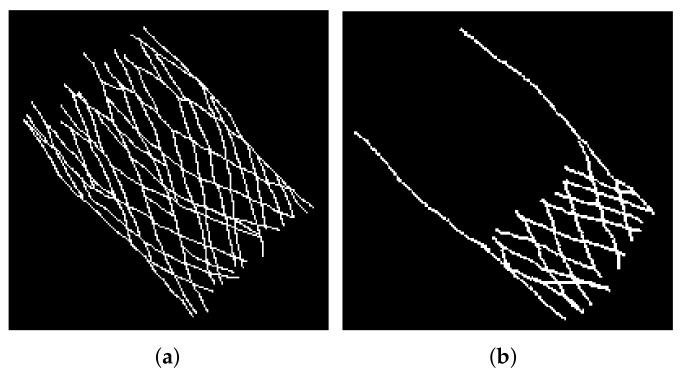
Cropped versions, only the region where the THV is contained, of the gold standard of different frame types. (**a**) Fully expanded THV. (**b**) Contrast injection.

Each of these images has been annotated, generating a gold standard. This is a set of labels of the frames containing the THV structure, an example can be seen in Figure 3a. These labels where manually drawn by the clinical members of the research team, namely, experienced hemodynamic cardiologists, using GNU Image Manipulation Program (GIMP) software. A particular case in the generation of the gold standard occurs in contrast injection frames, where the structure is only visible in part of the frame, as the threads cannot be distinguished where the constrast is injected. The gold standard of these frames considers as foreground only the part where the THV is visible, excluding the contrast pixels, yielding a partial pattern, as shown in Figure 3b.

### 3.2. Hyperparameter Tuning

As there are several hyperparameters and operation conditions of the architecture that play an important role in the quality of the segmentation results, a hyperparameter tuning stage is included in the methodology to study their repercussion and select an adequate configuration for this TAVI dataset. These hyperparameters considered for tuning the network are training set size, the use or not of data augmentation, and the cost function used in the model training.

Concerning the cost functions, two different options have been contemplated, namely, binary cross-entropy (2) and focal loss (4). For the selection of the focal loss parameters, the methodology proposed in the original focal loss work [16] in the ablation experiments for RetinaNet detector has been replicated, now using TAVI dataset and a U-Net. The best THV segmentation results have been obtained with α=0.25 and γ=2. On the other hand, the tuning stage considered data augmentation during training, applying transformations such as translation, zoom, rotation, and horizontal flip when it is enabled.

Different combinations of such hyperparameters have been tested, as will be detailed in following experiments, allowing to determine a proper configuration for the present problem and dataset by taking into account their evaluation metrics results.

### 3.3. THV Segmentation

The U-Net implementation used in this work for the segmentation of the THV has 64 filters in the first convolutional layer, as in the original paper [15], and it has been trained employing Adam optimizer [24].

The expected inputs are 512 × 512, 8-bit (integer values from 0 to 255), grayscale images and their corresponding labels as binary masks for the training. The outputs are 512 × 512 matrices representing the predicted probability of each pixel to belong to the foreground. Therefore, all the elements in the output images are within the interval [0, 1], but generally not binary. It is important to remark that these results do not label the frames in classes, they provide probability maps of this classification, and thus are called predictions. Binarized versions of such predictions would be called segmentations.

**Figure 4:**
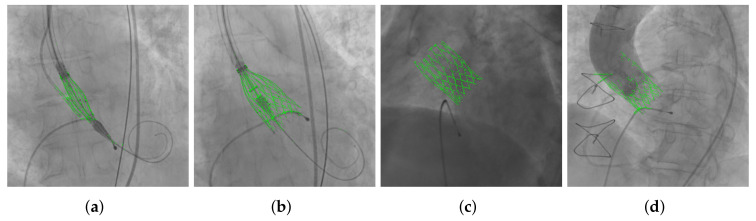
Different types of frames with their segmentation results (overlaid in green) obtained using Otsu’s method. (**a**) THV loaded in the deliverery catheter, previous to the deployment. (**b**) THV deployment. (**c**) THV totally deployed and expanded. (**d**) THV with contrast agent injection.

Figure 4 provides examples of segmentation results, as binarized versions obtained using Otsu’s method [25], overlaid on the original frames. The whole sequence from which the frame in Figure 4c was extracted is provided as a Supplementary Figure S2 (seg.gif), with the segmentation results overlaid.

### 3.4. Evaluation of the Segmentation Results

In the evaluation block, the quality of the results is assessed with different metrics by comparing each frame with the corresponding gold standard.

As the foreground in the gold standard consists of less than 1% of the image pixels, THV identification is a class imbalance problem. Therefore, conventional classification metrics used in supervised learning, based on the number of true/false positives and negatives, are not fully suitable on this problem [26]. Aiming to find a better way to assess the results, new metrics are required and will be defined. The first proposed metric, denoted as $D_{1}$, measures the average minimum Euclidean distance (in pixels) between the non-null elements in the gold standard and the closest non-null elements in the segmentations. It can be computed as follows:

(7) $$D_{1}\left(G,S\right)=\frac{1}{K}\sum_{g\in G}\min_{s\in S}d\left(g,s\right),$$

where *G* and *S* are the sets of non-zero elements in the gold standard (G) and a segmentation (S), respectively. *g* and *s* iterate over the points of such sets and d(·) denotes the Euclidean distance, measured in pixels, between two points. $\frac{1}{K}$ is a normalization term, being *K* the size of *G*, and this is the number of foreground pixels in the gold standard. In case of optimal segmentation, $D_{1}$ would be zero. However, notice that in an extreme case where the segmentation is an all-ones matrix, the distance would still be zero although the segmentation would be incorrect. In order to penalize this effect, a second metric $D_{2}$ is defined. $D_{2}$, expressed as (8), measures the noise by aggregating the probability in all the prediction pixels not present in the ground truth.

(8) $$D_{2}\left(G,P\right)=\sum_{g^{\prime }\in G^{c}}P_{g^{\prime }},$$

where *G* is the set of non-zero elements in the gold standard frame G, and $G^{c}$ is the complement of such set. Therefore, $G^{c}$ is the set of zero elements in G, and $g^{\prime }$ iterates over those points. $P_{g^{\prime }}$ is the element of the prediction frame P indexed by $g^{\prime }$. $D_{2}$ is inspired on false positive ratio, which is defined for binary classifications, whereas $D_{2}$ is computed on probability maps, not binary images. The combination of these two metrics $D_{1}$ and $D_{2}$ could show deeper insights about the results than other conventional metrics.

Some evaluation metrics used in this work require binary images as inputs, namely, two classes corresponding to background and foreground (the THV, in this case) in the scene. As the U-Net outputs provide probability maps, it is necessary to post-process these predictions to get binary images before computing the metrics. Such binarization can be performed in different ways and, consequently, provide different results. The first option considered was a fixed threshold value of 0.5. This approach has been tested, obtaining in general a number of foreground pixels under the average value known from the gold standard, as the probability values are often below such threshold. Considering other possibilities, one widely accepted approach is Otsu’s [25] method, which is used to perform automatic image thresholding. An alternative binarization proposed in this work is based on the prior knowledge of the foreground pixels (PKFP). The number of foreground pixels can be estimated as the mean number of foreground pixels in the set of gold standard images, obtaining 2346.04 ± 371.45 in mean and standard deviation from the frames where the THV is fully expanded. This estimation allows to build a binarization method by setting a threshold that assigns to foreground class the 2500 pixels with the highest probability values in the predictions.

## 4. Experiments and Results

Several experiments have been conducted to evaluate the performance of the proposed methodology, as the evaluation of different U-Net configurations, the assessment of the prediction results for the model trained with the best configuration obtained in the previous experiment.

This work includes 15 patients, 50 different angiographic sequences and a total of 2827 frames. The mean age of the whole population was 84.39 ± 4.02 years and an 83.33% were females.

### 4.1. Hyperparameter Tuning for the Model Selection

In order to identify an appropriate U-Net configuration, several architecture implementations and operating conditions were considered and evaluated. The best model obtained in this evaluation will be used in a posterior deeper analysis.

To perform this task, the dataset was split into two sets, training and test, with a ratio of 80/20. Such sets were generated by random selections, ensuring that the frames used to test the model did not belong to the patients used during training. The training set consisted of 2026 frames where the THV was completely expanded and 267 frames containing the aortic root previous to the device deployment, without any THV. Therefore, the split generated a training set constituted by a total of 2293 frame-label pairs extracted from 40 angiographic sequences and, on the other hand, a test set made of 534 images, obtained from 10 sequences.

Training set size is one of the parameters which could have important effects on the training process. To study its repercussion on performance, different subsets were generated from the training set with 600, 300, 150, and 75 images each. The subsets were generated with random selections from bigger subsets, such that each of them is contained in the subsets of greater sizes.

**Figure 5:**
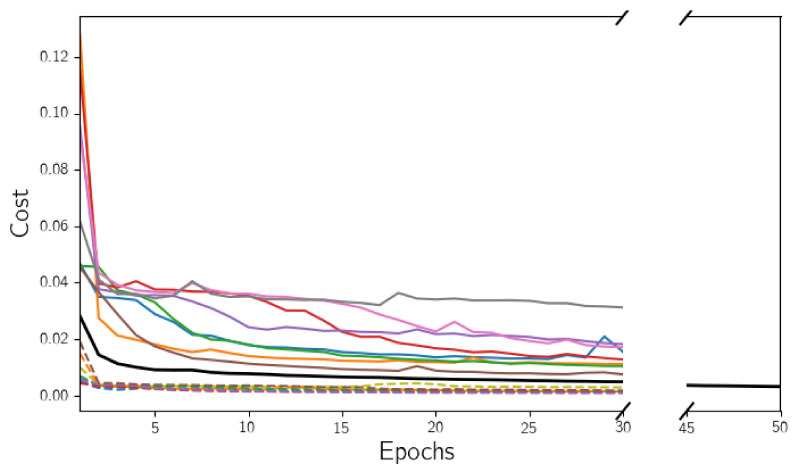
Cost function evolution with training epochs of all the models. Continuous lines correspond with the models using binary cross-entropy as cost function and dashed lines, with focal loss models. The black curve is the cost function of the best model, used in the evaluation in Section 4.2.

Experiments were conducted with binary cross-entropy (1) or focal loss (4) as cost functions, as well as using or not data augmentation, yielding four possible configurations for each dataset. As there are four different datasets, it makes a total of 16 models studied in this evaluation. All these models were trained for 30 epochs, using a batch size of 2 and Adam optimizer with a learning rate of $10^{-4}$. Figure 5 shows cost function evolution with training epochs of all the models used in this work, this being the 16 models considered in this evaluation and another one (black curve), which will be detailed in later sections. In all cases, it is observable the assymptotical behavior of the cost function. It can also be seen that focal loss values are always lower than binary cross-entropy, even from the first epoch.

Once the 16 models were trained, they were tested with the same test set, in order to obtain comparable results. This subset of images to test the models includes the 460 with fully expanded THV frames, extracted from the test set. The performance was evaluated in terms of $D_{1}$ (7) and $D_{2}$ (8), both metrics were computed for all the frames in the subset and expressed in mean and standard deviation values. All the results obtained in this evaluation of the U-Net configuration are provided in Table 1, where each row gathers the results of each tested configuration.

In this table, both cross-entropy (CE) and focal loss (FL) are considered, and the activation or not of data augmentation is expressed with a binary variable, DA∈{0,1}. Two measurements of $D_{1}$ are provided for each configuration, one where the binarization was obtained by Otsu’s method and the other, using PKFP binarization. The table also includes the best loss value achieved during the training of each model and the number of the training epoch where such value has been obtained. Loss values are not comparable for different cost functions since their typical values are not in the same range. However, analyzing separately the configurations using each cost function, it can be observed that there is a relation between loss values and the other metrics, as in general the models with the lowest loss values obtain the best results in terms of $D_{1}$ and $D_{2}$.

When a model obtains a high $D_{1}$ mean value, in many cases it is because the segmentations have few foreground pixels, and therefore tend to get low $D_{2}$ in average, as happens with the model trained with the dataset of 75 frames, data augmentation disabled, with cross-entropy as cost function, and using Otsu’s method for the binarization (first row in Table 1). On the other hand, a low $D_{1}$ value can be due to a high number of foreground pixels in segmentations, yielding high $D_{2}$ values as the model trained with the dataset of 75 frames, data augmentation enabled, with focal loss as cost function, and using Otsu’s method for the binarization (fourth row).

**Figure 6:**
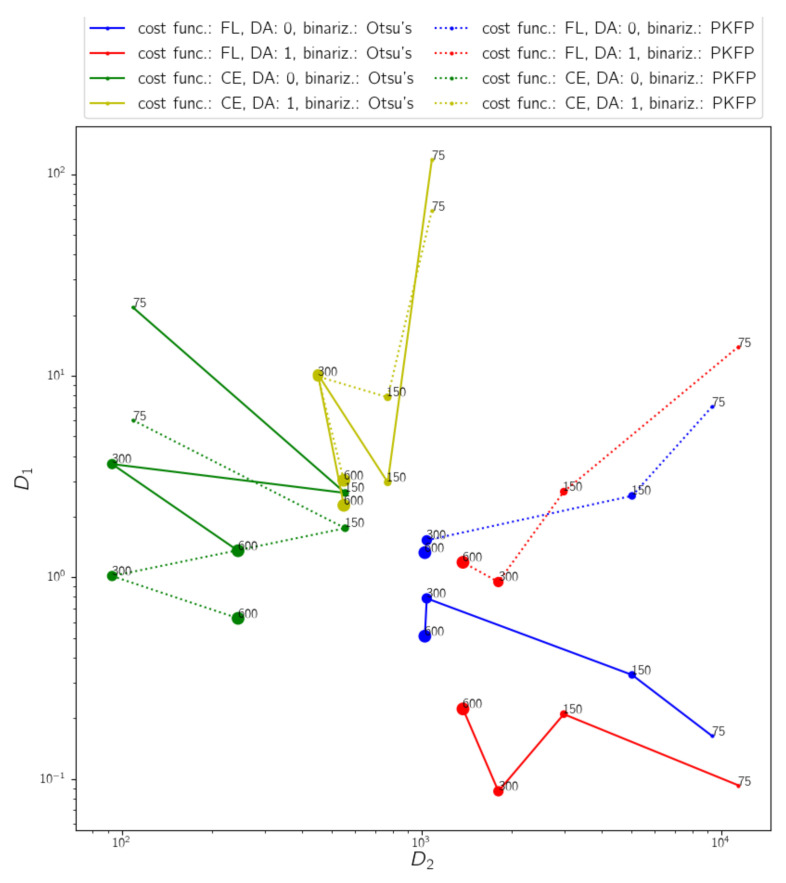
Graphical representation of the results in terms of $D_{1}$ and $D_{2}$ for each of the 16 models in Table 1. Each color corresponds to each of the four tested configurations. Blue: FL, DA=0; Red: FL, DA=1; Green: CE, DA=0; Yellow: CE, DA=1. Marker size refers to the number of images used to train each model (from 75 to 600). Continuous line $D_{1}$ values were computed using Otsu’s method, whereas discontinuous lines were obtained using PKFP binarization.

Figure 6 shows graphically the results from Table 1, both axes are in logarithmic scale for representation purposes. Each dot corresponds to a model and the coordinates are their $D_{1}$ and $D_{2}$ obtained mean values. Each line represents the evolution in performance of each configuration when the training set size increases, represented with an increase in the marker size. Continuous lines were obtained by computing $D_{1}$ on predictions binarized using Otsu’s method, and discontinuous lines correspond to PKFP binarization. $D_{2}$ is the same, as it is computed over the predictions and not the binary segmentations, therefore the difference between continuous and discontinuous analogous lines is a shift in $D_{1}$.

Regarding the results, in all cases the performance in terms of these metrics improves with training set size. It can be observed that in general the use of data augmentation does not provide an improvement in this problem. In addition, it is noticeable that binary cross-entropy configurations performed better than focal loss ones with this dataset.

**Figure 7:**
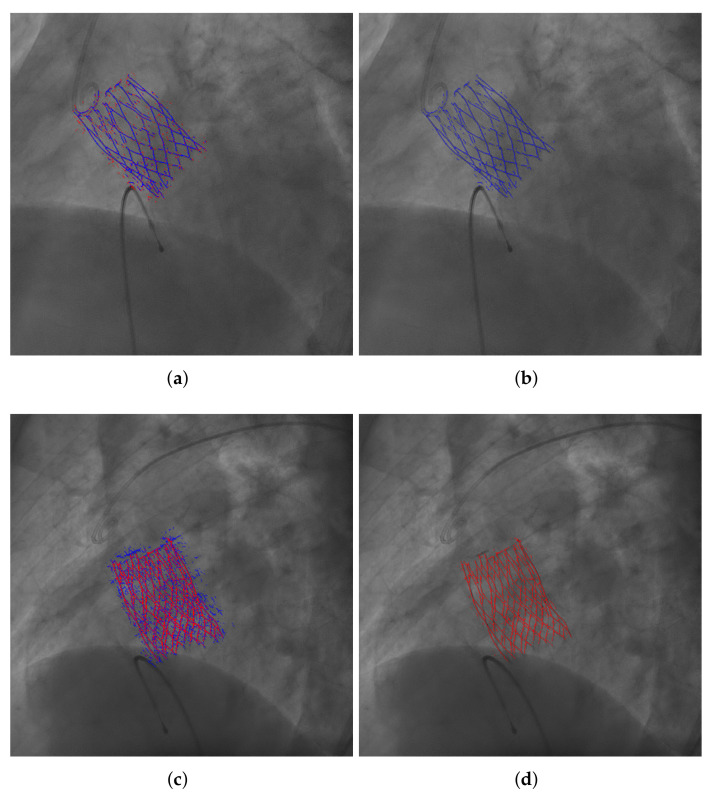
Examples of segmentation results obtained by different models, using Otsu’s method (blue) and PKFP (red) binarizations, overlaid on the original angiographic frames. (**a**) Segmentation obtained by the model trained with 600 frames, using cross-entropy and data augmentation disabled (13th row in Table 1), binarized using Otsu’s method and PKFP. (**b**) Same as (**a**), but with only Otsu’s method result overlaid. (**c**) Segmentation obtained by the model trained with 600 frames, using focal loss and data augmentation enabled (16th row in Table 1), binarized using Otsu’s method and PKFP. (**d**) Same as (**c**), but with only PKFP result overlaid.

The values of $D_{1}$ and other metrics are importantly affected by the binarization method. The suitability of such binarization on the predictions of a model is related to the cost function used during the training, as the predictions obtained by each cost function have their own distinctive properties. It has been observed that cross-entropy models tend to obtain incomplete patterns, whereas focal loss models usually produce noisy predictions. Directing attention to focal loss lines in the graph, Otsu’s binarization obtains lower $D_{1}$ values and appears to perform better, which is also noticeable in the segmentation examples in Figure 7a,b. However, this is because the binarization assigns many pixels to foreground, reducing $D_{1}$ but, at the same time, obtaining false positives. For focal loss models, PKFP actually obtains binarizations more similar to the ground truth than Otsu’s method, as can be seen in Figure 7c,d. On the other hand, as cross-entropy often gets false negatives, excluding pixels in the pattern, forcing a number of foreground pixels in the binarizations that might be too many, yielding binarizations with many false positives, as shown in Figure 7c. Otsu’s method performs better than PKFP in the segmentations of models trained using cross-entropy.

Regarding the results in terms of $D_{1}$ and $D_{2}$ in the performed experiments (results provided in Table 1), and normalizing by the worst mean result obtained for each of the metrics, the best configuration would be the closest to the origin. In this case, the best tested configuration with this dataset is using binary cross-entropy as cost function and disabling data augmentation. In addition, the model segmentation performance improves as the number of training samples grows.

### 4.2. Evaluation of the Results of the Selected Model

The best parameter configuration obtained in the previous section was used to train a new model, now using the whole training set, which is a total of 2293 frames. In addition, the number of training epochs was increased to 50 to get better parameter estimations. The black curve in Figure 5 shows the evolution of the cost function values obtained in each training epoch.

Several evaluations and experiments were conducted, assessing the model on different subsets of images, all of them belonging to the test set, not included in the training. Table 2 gathers the results of all the performed experiments, where each column in the table corresponds to an evaluation. The metrics are computed over several frames and the results are expressed in mean and standard deviation for different image sets. For metrics requiring binary inputs, the results are computed on images binarized using both Otsu’s method and PKFP. The number of test frames used in each experiment is also specified in the table.

The first experiment evaluates the U-Net model on frames where the THV is fully expanded, computing the metrics on the 460 frames in the test set; an example of the obtained results is shown in Figure 4c. Comparing these results with the ones obtained by the best configuration in the previous section, which was tested on the same set of images, it can be observed an improvement in $D_{1}$ and $D_{2}$, an effect of the increase in the training set size and the number of training epochs.

The second experiment analyzes the model performance on 65 frames belonging to the test set, containing the aortic root before the deployment, where the THV is not present. $D_{1}$ cannot be obtained, as there is not any pattern to identify. Furthermore, AUROC is not defined for ground truths where only one class is present, and precision, recall, Dice coefficient, and Jaccard index are equal to 0, independently of the correctness of the segmentation. $D_{2}$ is specially insightful in this scenario, obtaining a rather low value, indicating that the model performs well in these frames. It should be noted that, even though it is not reflected in the metrics, PKFP binarization is not opportune in this set of images, as the optimum segmentation would not include any foreground pixels.

The third experiment evaluates the methodology performance to identify the THV during its deployment using three frames, obtaining segmentations as the example in Figure 4b. Results in terms of some metrics are significantly worse than previous experiments. It must be taken into account that the device shape during deployment is not that similar to the bioprosthesis fully deployed and the model has not been trained to identify those different shapes. This fact makes these images harder to segment and the results are often incomplete.

The fourth experiment addresses the effect of injecting contrast, evaluating six frames, an example of a segmentation result is shown in Figure 4d. Even though the model identifies part of the THV correctly, $D_{1}$ and $D_{2}$ are impaired due to the contrast part, where significant errors occur.

All the experiments achieve high accuracy values. This occurs because almost all background pixels are correctly classified which, given the strong class imbalance, entails a great part of the frame. All the experiments show a poor performance in terms of Dice coefficient, which can be explained by the fact that this metric measures the overlapping between areas, but the THV in the frames is a thread-like structure. The experiments have shown the important impact of the binarization method on the results, obtaining noticeable differences in all cases, except accuracy. Precision and Jaccard index get better results using Otsu’s method, whereas PKFP binarization improves recall and Dice coefficient results.

## 5. Discussion

The automatic interpretation of angiographic images, if obtained in an unattended manner, can provide valuable additional information during TAVI. The extraction of this information usually may rely on the automatic detection of the bioprosthesis in the image sequence. This work presents a fully automatic way of identifying and segmenting THV on angiographic sequences, training a U-Net and studying different configurations aiming to improve the segmentation performance. Given an annotated dataset of TAVI angiographies, including frames of different stages of the procedure, the method has demonstrated its performance with AUROC values between 0.85 and 0.98 in the experiments.

Validation is of main importance in this kind of applications. Finding the right metrics and assessing the quality of the results can be more difficult than obtaining the solution itself. In this work, standard metrics based on pixel-wise evaluation are used, and new metrics are specifically proposed for this problem. The defined $D_{1}$ and $D_{2}$ measure more appropriately the similarity between segmentations and the gold standard. The binarization also has an impact on validation, as several metrics require binary inputs. Using a threshold of 0.5 did not perform correctly; thus, other schemas have been considered, analyzing their suitability for the problem. Both Otsu’s method and the defined PKFP binarization have demonstrated their validity; however, it has been observed a relation with the cost function used during the model training: Otsu’s method is the prefered option for cross-entropy, whereas focal loss models require PKFP binarization. Notice that such PKFP binarization requires a priori knowledge of the THV and it is only valid when the device is present, not being suitable on angiographic frames previous to the delivery.

Concerning the benefits for the clinical practice, the main contribution of this work relies on the possibility of using TAVI segmentation on angiographic sequences, automatically delineating the THV. This work is a first step that would permit the extraction of quantitative features from the images, which could provide several advantages as reducing subjectivity of the physicians during the procedure to get the correct placement of the bioprosthesis or check the device expansion in real-time. The latter may help physicians to decide objectively the need for conducting different measures during TAVI to obtain optimal results (balloon post dilatation due to THV infra expansion o significant paravalvular leakeage), and ensure better long-term outcome. Furthermore, the information that could be extracted from the sequences, if obtained in real time, could lead to the automation of the procedure. However, this is a major challenge to be explored, beyond the scope of this paper.

The methodology presented in this work has proved success in the automatic identification of Allegra THV on X-ray angiographic sequences. However, it must be taken into account that this method could also be used to identify other THV models and devices from other manufacturers. Even more, the methodology could be applied in the general problem of identification of ridge structures in X-ray sequences, extending significantly the field of application.

## 6. Conclusions

This paper presents an automatic method for detecting the THV prothesis on angiographic sequences using deep learning techniques, without the need of any human interaction. The methodology relies on a U-Net architecture, including a hyperparameter tuning stage for the selection of a proper configuration, and an evaluation of the obtained segmentation results. Such evaluation considers different conventional metrics as well as two new ones, defined for this specific purpose, in addition to the study of the impact of different binarization schemas in the metrics results. The models are trained using an annotated dataset and tested in different kind of frames captured during TAVI procedure, showing satisfactory results.

## Figures and Tables

**Table 1:** Results of the 16 models considered in the evaluation of the U-Net configuration.

Training Set Size	Data Aug. (DA)	Loss Function	Best Epoch	Best Loss Value	$D_{1}$ (Otsu’s Binarization)	$D_{1}$ (PKFP Binarization)	$D_{2}$
75	0	CE	30	0.0171	21.87 ± 26.43	6.03 ± 3.79	108.79 ± 81.54
		FL	6	0.0028	0.16 ± 0.55	7.02 ± 3.75	9314.98 ± 3149.51
	1	CE	30	0.0312	118.38 ± 218.96	65.96 ± 56.89	1082.49 ± 666.88
		FL	30	0.0028	0.093 ± 0.40	13.95 ± 8.07	11415.52 ± 3787.17
150	0	CE	30	0.0128	2.62 ± 3.69	1.75 ± 2.11	556.16 ± 232.48
		FL	30	0.0015	0.33 ± 0.56	2.54 ± 1.45	5041.36 ± 1334.57
	1	CE	30	0.0182	2.96 ± 4.87	7.82 ± 8.99	771.07 ± 313.18
		FL	30	0.0016	0.21 ± 0.42	2.66 ± 1.73	2983.14 ± 1011.65
300	0	CE	30	0.0085	3.65 ± 3.15	1.02 ± 1.07	92.81 ± 62.13
		FL	29	0.0010	0.79 ± 2.03	1.53 ± 2.49	1040.91 ± 220.49
	1	CE	26	0.0129	10.16 ± 17.12	9.92 ± 13.54	451.25 ± 161.92
		FL	30	0.0012	0.09 ± 0.18	0.95 ± 0.97	1804.71 ± 353.82
600	0	CE	30	0.0075	1.36 ± 1.56	0.63 ± 0.73	244.23 ± 90.97
		FL	30	0.0009	0.51 ± 3.40	1.33 ± 4.63	1025.85 ± 170.54
	1	CE	29	0.0112	2.28 ± 4.82	3.03 ± 5.02	550.40 ± 176.58
		FL	30	0.0012	0.22 ± 0.37	1.19 ± 1.14	1374.78 ± 233.04

**Table 2:** Results of the selected model in the experiments.

Metric	Bin. Method	Exp. 1 (460 Frames)	Exp. 2 (65 Frames)	Exp. 3 (3 Frames)	Exp. 4 (6 Frames)
$D_{1}$	Otsu’s	0.91 ± 0.86	–	67.95 ± 75.11	10.21 ± 8.73
	PKFP	0.30 ± 0.38	–	52.94 ± 62.72	3.92 ± 4.16
$D_{2}$	–	138.48 ± 75.76	2.40 ± 9.21	204.45 ± 19.83	369.83 ± 48.50
Accuracy	Otsu’s	0.99 ± 0.01	0.99 ± 0.01	0.99 ± 0.01	0.99 ± 0.01
	PKFP	0.99 ± 0.01	0.99 ± 0.01	0.99 ± 0.01	0.99 ± 0.01
Precision	Otsu’s	0.73 ± 0.05	0	0.51 ± 0.06	0.46 ± 0.06
	PKFP	0.63 ± 0.07	0	0.43 ± 0.06	0.34 ± 0.04
Recall	Otsu’s	0.87 ± 0.04	0	0.70 ± 0.09	0.75 ± 0.04
	PKFP	0.97 ± 0.02	0	0.80 ± 0.09	0.93 ± 0.03
Dice Coefficient	Otsu’s	0.15 ± 0.03	0	0.29 ± 0.05	0.33 ± 0.04
	PKFP	0.22 ± 0.06	0	0.36 ± 0.05	0.47 ± 0.06
Jaccard Index	Otsu’s	0.74 ± 0.04	0	0.55 ± 0.06	0.50 ± 0.05
	PKFP	0.64 ± 0.07	0	0.47 ± 0.06	0.36 ± 0.03
AUROC	Otsu’s	0.94 ± 0.02	–	0.85 ± 0.05	0.89 ± 0.02
	PKFP	0.98 ± 0.01	–	0.90 ± 0.04	0.96 ± 0.01

## Data Availability

Data not publicly available due to privacy restrictions, but available from the corresponding author C.V., upon reasonable request.

## Supplementary Materials

The following supporting information can be downloaded at: https://www.mdpi.com/article/10.3390/diagnostics12020334/s1, Figure S1: angioseq.gif. Figure S2: seg.gif.
