# Supplementary figures and images for: Automatic Identification of Bioprostheses on X-ray Angiographic Sequences of Transcatheter Aortic Valve Implantation Procedures Using Deep Learning
Source: Diagnostics (Basel). 2022 Jan 27;12(2):334. doi: 10.3390/diagnostics12020334 (PMC8870761; doi:10.3390/diagnostics12020334)

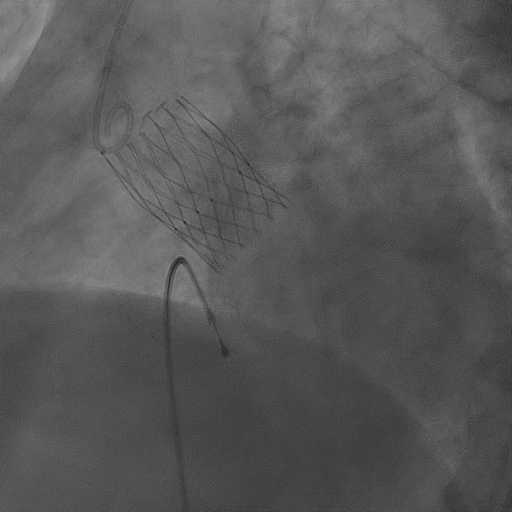

Supplement: Supplementary file 1 [file diagnostics-12-00334-s001.zip › diagnostics-1544493-supplementary/Figure S1. angioseq.gif]

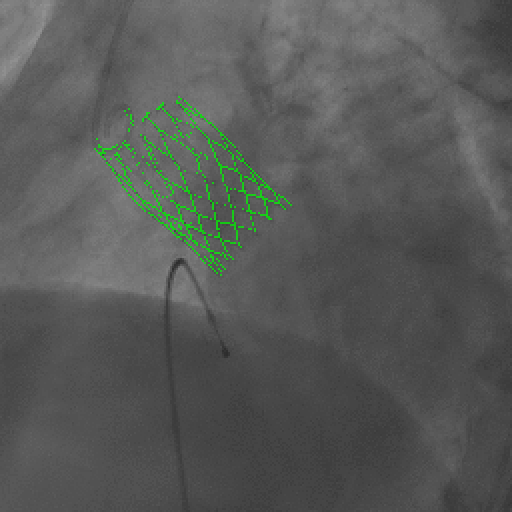

Supplement: Supplementary file 1 [file diagnostics-12-00334-s001.zip › diagnostics-1544493-supplementary/Figure S2. seg.gif]
